# Potential Antiglycation and Hypoglycaemic Effects of* Toona ciliata* M. Roem. and* Schkuhria pinnata* Lam. Thell. Crude Extracts in Differentiated C2C12 Cells

**DOI:** 10.1155/2019/5406862

**Published:** 2019-01-21

**Authors:** Brian K. Beseni, Thabe M. Matsebatlela, Victor P. Bagla, Idris Njanje, Kgomotso Poopedi, Vusi Mbazima, Leseilane Mampuru, Matlou P. Mokgotho

**Affiliations:** University of Limpopo Department of Biochemistry, Microbiology and Biotechnology, Private Bag x1106, Sovenga 0727, South Africa

## Abstract

Medicinal plants have been identified as a feasible avenue for the development of new potent antidiabetic agents. The phytoconstituent compositions of different* Toona ciliata *and* Schkuhria pinnata *extracts were determined and quantified using standard chemical methods after exhaustive extraction. Thereafter, their antioxidant and antiglycation potentials were spectrophotometrically determined. The cytotoxicity profiles of the extracts on C2C12 cells were determined using the MTT assay.* Toona ciliata* methanol extract resulted in the highest percentage yield (20.83%) and high total phenols and flavonoids content in the methanol and acetone extracts compared to* S. pinnata* extracts. The acetone extract of* T. ciliata* showed good activity in the DPPH scavenging and FRAP assays with EC_50_ values of 1.90 mg/ml and 5.26 mg/ml, respectively. Arbutin's antiglycation ability was outperformed by treatments with the methanol, acetone, and hexane extract of* T. ciliata *which resulted in 2.49%, 2.79%, and 2.56% glycation, respectively. The hexane extract of* T. ciliata *was less toxic to C2C12 cells as compared to the other extracts with CC_50_ value of 402.16 *μ*g/ml. Only the hexane extract of* S. pinnata *resulted in glucose utilisation of 28.56% which was higher than that of insulin (26.06%) after 6 hours and is therefore considered as the most potent extract with hypoglycaemic potential in this study. Studies are ongoing aimed at identifying drug candidates in this extract that may be employed in the development of hypoglycaemic, antioxidant, and antiglycation agents.

## 1. Introduction

Diabetes mellitus is a noncurable, multifactorial, and noncommunicable condition with symptoms that clinically manifest as perpetual hyperglycaemia [1, 2]. Prolonged hyperglycaemia can lead to accelerated rate of glycation in diabetic patients as opposed to individuals with normal postprandial glucose levels [3, 4]. Glycation, which occurs via the Maillard reaction, is the spontaneous reaction between structural or functional proteins and reactive sugar moieties [5]. This results in the formation of advanced glycation end-products (AGEs) which are essentially accumulated glycosylated proteins [4, 6]. Glycosylated proteins are known to have disrupted molecular conformation, altered enzymatic activity, and reduced degradative capacity and interfere with various receptor recognition processes [7]. In diabetes, the most apparent product is glycated haemoglobin A_1c_ (A1C) [6]. Glycation is known to be the underlying cause in both the development and perpetuation of the complications associated with diabetes [5]. These complications include neuropathy, nephropathy, retinopathy, and various cardiovascular diseases [2, 4]. These complications lead to infinite debilitating signs such as blindness, amputations, and premature deaths among those people living with diabetes. Three main etiological types of diabetes are prominent which are type I, type II, and gestational diabetes [2].

The prevalence of diabetes, particularly type II which is associated with lifestyle, continues to rise and has reached global epidemic status [8]. The International Diabetes Federation (IDF) projects that by the year 2040 the global prevalence of diabetes will rise to an alarming 1 in 10 adults which translates to about 641.7 million people [9]. This high prevalence comes with a high economic burden to both the families of affected individuals and countries at large. Majority of countries spend between 5% and 13% of their total health expenditure on diabetes [10]. At such a high cost, the disease is a significant challenge for healthcare systems and an obstacle to sustainable economic development. It is projected that the global health expenditure on diabetes will rise from at least USD 376 billion in 2010 to approximately USD 490 billion in the year 2030 [10]. The fact that over 74% of the current western pharmacological therapeutic drugs are derived from medicinal plants has sparked interest in the investigation of these medicinal plants for the development of new effective drugs derived from these plants [11, 12].* Toona ciliata *and* S. pinnata *were investigated in this study as they have been reported to have antidiabetic activities among other therapeutic functions [13–17].

*Schkuhria pinnata *(Lam.) Thell. of the family Asteraceae is commonly known as the dwarf marigold. In Kenya, the whole plant is burnt, and water is added to the ashes and the resulting infusion is taken orally for diabetes management [13].* Toona ciliata*, commonly known as the red toon, belongs to the family Meliaceae. Several compounds have been isolated from this plant including siderin and cedrelone which are known to have very potent antioxidant activity [14]. Various parts of the plants are used for different purposes; the leaves have been reported to have hypoglycaemic, spasmolytic, and antiprotozoal activity [15], while on the other hand the barks are used as antiulcer and for menstrual disorders, respectively [16]. Apart from a report by Rana [17] showing* T. ciliata* to possess antihyperglycaemic activity in streptozotocin induced diabetes in rats, its antidiabetic modes of action remain poorly studied and understood. This study was aimed at determining the antiglycation, antioxidant, and hypoglycaemic activity of the selected plants to validate their traditional use.

## 2. Materials and Methods

### 2.1. Plant Collection and Verification

Leaves of* T. ciliata. *and* S. pinnata *were collected from Mankweng area, Capricorn Local Municipality, Limpopo Province, South Africa. The plants were selected based on literature surveys of reports on their antidiabetic properties by traditional healers and village elders in the Limpopo Province. The identities of the plants were authenticated by Dr B Egan, a curator at the Larry Leach Herbarium, University of Limpopo, and accorded voucher specimen numbers:* T. ciliata *(UNIN 12331) and* S. pinnata *(UNIN 121066).

### 2.2. Plant Extract Preparation

Air-dried leaf materials used in the study were ground into a fine powder using a domestic warring blender. Powdered materials (1 g) were exhaustively extracted using 10 ml each of methanol, acetone, and hexane [18]. The supernatants were filtered using a Whatman No.1 filter paper into preweighed glass vials and air-dried under a stream of cold air. The quantities extracted were determined and samples stored in air-tight glass vials in the dark until use. The dry plant extracts were reconstituted in Dimethylsulphoxide (DMSO) (Sigma Aldrich™, SA), distilled water, or acetone where appropriate for the different assays.

### 2.3. Determination of Secondary Metabolites

The presence of different plant secondary metabolites in the crude extracts was determined using various standard chemical tests as described by Harborne [19].

### 2.4. Total Phenolic Content

The total phenolic contents of the different extracts were determined spectrophotometrically using Folin-Ciocalteu's phenol reagent method [20]. Stock solutions (100 mg/ml) of the different extracts were prepared. Folin-Ciocalteu reagent (50 *μ*l) and 450 *μ*l distilled water were added to 100 *μ*l of each of the extracts (1 mg/ml) and allowed to stand for 5 minutes in the dark at room temperature. Thereafter, 500 *μ*l of a 7% sodium carbonate solution was added. Distilled water was added to make a final volume of 5000 *μ*l and the mixture allowed to stand for 90 minutes in the dark at room temperature. Absorbance of the mixture in triplicate was measured at 750 nm using a spectrophotometer (Beckman Coulter-DU730). The total phenolic content was determined by linear regression from a tannic acid calibration standard curve.

### 2.5. Total Flavonoid Content

Aluminium chloride colorimetric method was used for determination of total flavonoids [21]. A stock solution of 10 mg/ml, each of the different extracts was prepared and 100 *μ*l mixed with 100 *μ*l of 10% aluminium chloride, 1 M potassium acetate (100 *μ*l), and 2800 *μ*l distilled water. The mixture was left to stand at room temperature for 30 minutes. The absorbance of the reaction mixture was measured at 415 nm in triplicate using a spectrophotometer (Beckman Coulter-DU730). The total flavonoid content was determined by linear regression from a quercetin calibration curve standard.

### 2.6. Determination of Antiglycation Activity

Antiglycation activities of the plant extracts were determined using the bovine serum albumin glycation end-products (AGEs) assay with slight modification [22]. Bovine serum albumin (BSA) (Sigma Aldrich) (500 *μ*l) was incubated with 400 *μ*l of glucose and 100 *μ*l of plant extracts (1 mg/ml). Phosphate buffer saline (100 *μ*l) was used as the sample control and 100 *μ*l Arbutin (Sigma Aldrich) as the reference standard. A negative control constituting of 500 *μ*l BSA, 400 *μ*l phosphate buffer saline, and 100 *μ*l plant extracts was included. The reaction mixture was allowed to proceed at 60°C for 72 hours and terminated by addition of 10 *μ*l of 100% (w/v) trichloroacetic acid (TCA) (Sigma Aldrich). The TCA added mixture was kept at 4°C for 10 minutes and thereafter centrifuged for 4 minutes at 13000 rpm. The precipitate was redissolved in alkaline phosphate buffer saline (pH 10) and quantified for relative amount of glycated BSA, based on fluorescence intensity in 96-well plates using a microtiter-plate multimode detector (Promega-Glomax Multi detection system). The excitation and emission wavelength used were at 370 nm and 440 nm, respectively. Five concentrations of each sample were analysed in triplicate. Percentage inhibition was calculated using the formula provided below and the sample concentration required for 50% inhibition of BSA glycation was calculated:

(1) %  inhibition=OD  blank−OD  sample−OD  sample  negativeOD  blank  x  100

### 2.7. Quantitative DPPH Radical-Scavenging Activity Assay

The antioxidant activity of each of the different extracts was quantitatively determined spectrophotometrically using the 2, 2-diphenyl-1-picrylhydrazyl (DPPH) free radical scavenging assay [23]. Equal volumes of 0.2% DPPH in methanol and different concentrations (0 *μ*g/ml to 1000 *μ*g/ml) of the extracts were incubated in the dark at room temperature for 30 minutes. The DPPH in methanol solution was used as the experimental control, and L-ascorbic acid (vitamin C) as a positive control. The decrease in absorbance was measured at 490 nm using a microtiter-plate multimode detector (Promega-Glomax, Multidetection system). The degree of discolouration indicates the scavenging potential of the extracts in terms of hydrogen donating ability. The absorbance values obtained were converted into percentage scavenging activity using the following formula:

(2) %  inhibition=A490nm  of  blank−A490nm  of  sample  x  100A490nm  of  blank

### 2.8. Ferric Reducing Antioxidant Power

The ferric reducing antioxidant power of the different extracts was determined [24]. Various concentrations ranging from 0 *μ*g/ml to 1000 *μ*g/ml of the extracts in deionised water (100 *μ*l) were prepared. A blank was prepared without extract, while ascorbic acid was used as the reference standard. These were then mixed with 250 *μ*l phosphate buffer (pH 7.4 and concentration 0.2 M) together with 250 *μ*l potassium ferricyanide and incubated at 50°C for 20 minutes. After incubation, aliquots of 250 *μ*l trichloroacetic acid were added to the mixture and centrifuged at 3000 rpm for 10 minutes. The supernatant (250 *μ*l) was mixed with 250 *μ*l distilled water and freshly prepared ferric chloride solution (50 *μ*l). The absorbance of the samples was measured at 700 nm using a microtiter-plate multimode detector (Promega-Glomax Multidetection system). Percentage reducing power was calculated according to the following formula:

(3) Percentage  reducing  power=A700nm  of  sample−1  x  100A700nm  of  blank

The effective concentration (EC_50_) values, which represent a concentration that elicit a 50% response, were determined by regression analysis, from linear plots of concentration of the extract against the mean percentage of the antioxidant activity.

### 2.9. Maintenance of Cell Culture

An immortalised mouse myoblast cell line C2C12 (ATCC, Rockville, USA) was used in this study. The cells were cultured and maintained in Roswell Park Memorial Institute medium (RPMI) media (Lonza, BioWhittaker®), supplemented with 10% FBS (Hyclone, Thermo Scientific) at 37°C, in an atmosphere of 5% CO_2_ in a humidified incubator (Heracell 150i CO_2_ incubator, Thermo Scientific). The cells were differentiated by culturing in RPMI media containing 2% horse serum for 4 days.

### 2.10. Cytotoxicity Assay

The cytotoxicity of the different plant extracts on C2C12 cells were determined using the 3-(4,5-dimethylthiazol-2-yl)-2,5-diphenyltetrazolium bromide (MTT) assay (Sigma Aldrich, SA) as modified by Ferrari and colleagues [25]. Experiments were done in triplicate in three independent trials. Cells were seeded at an initial cell density of 2 x 10^5^ cells/ml into 96-well cell culture plates (Nunc™, Roskilde, Denmark). The cells were incubated overnight to allow the cells to attach. The cells were untreated and treated with different concentrations (0 *μ*g/ml to 1000 *μ*g/ml) of the different extracts. The untreated cells served as the experimental control. Actinomycin (Sigma Aldrich, SA) and DMSO served as positive and negative controls, respectively. The plates were incubated at 37°C for 24 hours after which 10 *μ*l MTT (10 mg/ml) was added to each well. The cells were further incubated at 37°C for 2 hours. The medium was aspirated and the cells were washed once with prewarmed PBS, pH 7.4. The insoluble purple coloured formazan formed intracellularly by the action of the mitochondrial dehydrogenase of viable cells following reaction with MTT was solubilised using 100 *μ*l DMSO. The absorbance was measured at 490 nm using a microtiter-plate multimode detector (Promega-Glomax Multidetection system). The percentage of viable cells was calculated according to the following formula below:

(4) $$\begin{array}{c} Percentage\;\;viability=\frac{\left(A_{490nm}\;\;of\;\;sample\;\;x\;\;100\right)}{\left(A_{490nm}\;\;of\;\;control\right)} \end{array}$$

### 2.11. Glucose Uptake Assay

The amount of glucose taken up by differentiated C2C12 cells was quantified using the glucose uptake kit according to the manufacturer's instructions (KAT Laboratories and Medicals (PTY) LTD). Cells at an initial seeding density of 5x10^4^ were treated for 1, 3, and 24 hours in the presence or absence of the different plant extracts. Untreated cells were used as the experimental control, while insulin and DMSO were used as positive and negative controls, respectively. After treatment, the media (1 *μ*l of the supernatant) from each of the treatments, including the control, were transferred into new 96-well flat bottomed plates and 100 *μ*l working reagent was added and protected from light. The mixture was incubated in the dark at 37°C for 5 minutes. Absorbance at 500 nm was immediately read using a microtiter-plate multimode detector (Promega-Glomax Multidetection system). The results are presented as percentage glucose utilisation.

### 2.12. Linear Regression and Statistical Analysis

The results were obtained from three independent experiments run in triplicate and expressed as means ± standard deviation. The effective concentration (EC_50_) and cytotoxicity concentration (CC_50_) values which represent a concentration that elicit a 50% response were determined by regression analysis. The statistical significance of the results was tested using One-Way Analysis of Variance (ANOVA) employing the Dunnett Multiple Comparisons Test between the control and the different treatments within the same group and the Tukey-Kramer Multiple Comparisons Test. The* p* value significance is represented as asterisk (*∗*) for* p* <0,05, two asterisks (*∗∗*) for* p*<0,01, and three asterisks (*∗∗∗*) for* p*<0,001.

## 3. Results

### 3.1. Plant Material Extraction

**Figure 1:**
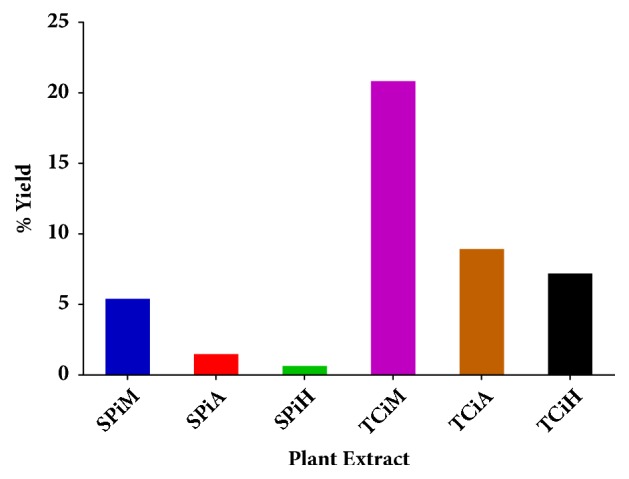
Percentage yields of the plant extracts obtained using solvents of varying polarity. SPiM=*S. pinnata *methanol extract, SPiA=*S. pinnata *acetone extract, SPiH=* S. pinnata* hexane extract, TCiM=*T. ciliata *methanol extract, TCiA=*T. ciliata* acetone extract, and TCiH=*T. ciliata *hexane extract.

The percentage yields of the different crude extracts obtained using solvents of varying polarity, namely, methanol, acetone, and hexane, are shown in Figure 1. Methanol had the highest extraction percentage yield while hexane had the least yield in both plants. The highest percentage yield was obtained for the methanol extract of* T. ciliata* (20.83%) and lowest for the hexane extract of* S. pinnata *(0.64%).

### 3.2. Secondary Metabolite Analysis

Qualitative analysis of the phytochemicals was performed in order to determine the presence of tannins, flavonoids, phenols, saponins, steroids, phlobatannins, glycosides, coumarins, proteins, anthraquinones, anthocyanins, leucoanthocyanins, turns, and carbohydrates in all the crude plant extracts. All the extracts of* S. pinnata *contained phenols and tannins and not saponins, phlobatannins anthraquinones anthocyanins, leucoanthocyanins, turns, and carbohydrates (Table 1). The methanol and acetone extracts tested positive for the presence of flavonoids, glycosides, coumarins, and proteins but absent in the hexane extract.* Toona ciliata* on the other hand tested positive for the presence of tannins, flavonoids, phenols, steroids, and coumarins and absent for saponins, phlobatannins, glycosides, proteins, anthraquinones, anthocyanins, leucoanthocyanins, turns, and carbohydrates (Table 1).

### 3.3. Quantitative Phenolic and Flavonoid Analysis

**Figure 2:**
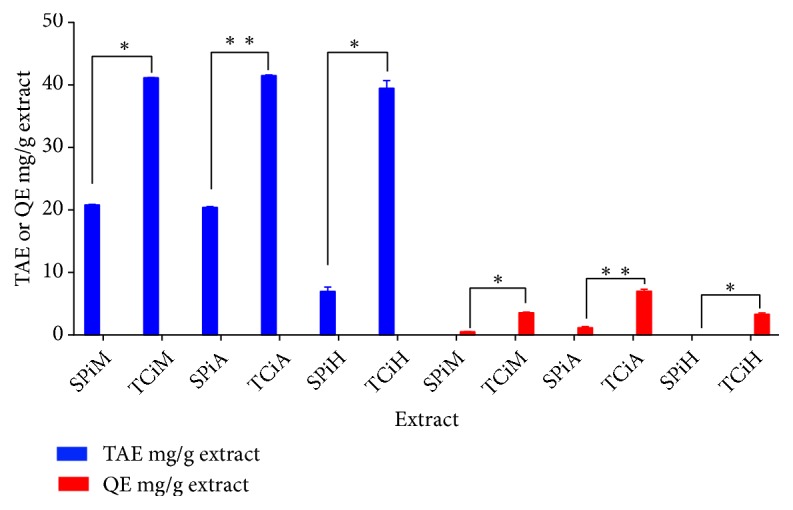
The total phenolic content of the different dry plant extracts represented as tannic acid equivalents (TAE mg/g) and flavonoids content in the different plant extracts represented as quercetin equivalents (QE mg/g). SPiM=*S. pinnata *methanol extract, SPiA=*S. pinnata *acetone extract, SPiH=*S. pinnata* hexane extract, TCiM=*T. ciliata *methanol extract, TCiA=*T. ciliata *acetone extract, and TCiH=*T. ciliata *hexane extract. The asterisks represent significant differences in the quantity of total phenolic content or flavonoids between the extracts of the same solvent from the different plants. The* p* value significance was represented by an asterisk (*∗*) for* p* <0,05 and two asterisks (*∗∗*) for* p* <0,01.

The flavonoid and total phenolic content of each of the extracts were determined as quercetin and tannic acid equivalents, respectively (Figure 2). The methanol and acetone extracts contained the highest amount of flavonoid and phenolic contents, while hexane extracts had the least in both plants, although* T. ciliata *extracts contained significant amounts of total phenolics and flavonoids compared to* S. pinnata* extracts.

### 3.4. Quantitative FRAP and DPPH

The EC_50_ values for DPPH scavenging assay and ferric reducing power of the different* T. ciliata *and* S. pinnata* extracts (Table 2). The acetone extract of* T. ciliata* showed the best activity among all the extracts in both the DPPH and FRAP assays. It exhibited EC_50_ values of 1.90 mg/ml and 5.26 mg/ml for the DPPH scavenging activity and the ferric reducing power assays, respectively. These EC_50_ values were however not lower than those for ascorbic acid (positive control) which were 1.62 mg/ml and 3.10 mg/ml for the DPPH scavenging activity and the ferric reducing power assays, respectively.

### 3.5. Antiglycation Activity

**Figure 3:**
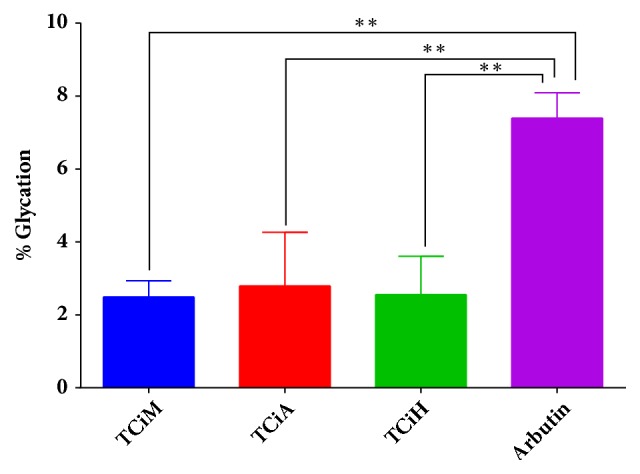
The effects of different extracts of* T. ciliata *on the glycation of bovine serum albumin (BSA). Arbutin was used as the standard reference. TCiM=*T. ciliata *methanol extract, TCiA=*T. ciliata *acetone extract, and TCiH=*T. ciliata* hexane extract. The results were obtained from three independent experiments and expressed as means ± standard deviation. The statistical significance of the results was tested using One-Way ANOVA employing the Tukey-Kramer Multiple Comparisons Test. The* p* value significance was represented by two asterisks (*∗∗*) for* p*<0,01.

The inhibitory effect of the extracts on bovine serum albumin glycation was conducted (Figure 3). The extracts of* T. ciliata *exhibited high antiglycation activity. Treatments with the methanol, acetone, and hexane extracts resulted in 2.49%, 2.79%, and 2.56% glycation activity, respectively. The antiglycation activities of all the extracts were significantly higher (*p*<0.01) than that of Arbutin (positive control).

**Figure 4:**
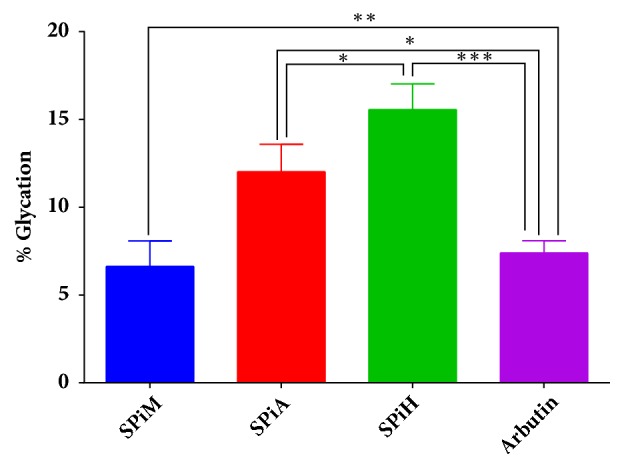
The effects of different extracts of* S. pinnata *on the glycation of Bovine serum albumin (BSA). Arbutin was used as the standard reference. SPiM=*S. pinnata *methanol extract, SPiA=*S. pinnata *acetone extract, and SPiH=*S. pinnata* hexane extract. The results were obtained from three independent experiments and expressed as means ± standard deviation. The statistical significance of the results was tested using One-Way ANOVA employing the Tukey-Kramer Multiple Comparisons Test. The* p* value significance was represented by an asterisk (*∗*) for* p* <0,05, two asterisks (*∗∗*) for* p* <0,01, and three asterisks (*∗∗∗*) for* p* <0,001.

The methanol extract of* S. pinnata* resulted in 6.62% glycation (Figure 4) which was significantly lower than the amount of glycated BSA after treatment with Arbutin (7.40%). On the other hand, the acetone and hexane extracts resulted in 12.02% and 15.56% glycation, respectively, which were not effective as compared to Arbutin (7.40%).

### 3.6. Cytotoxicity Analysis

**Figure 5:**
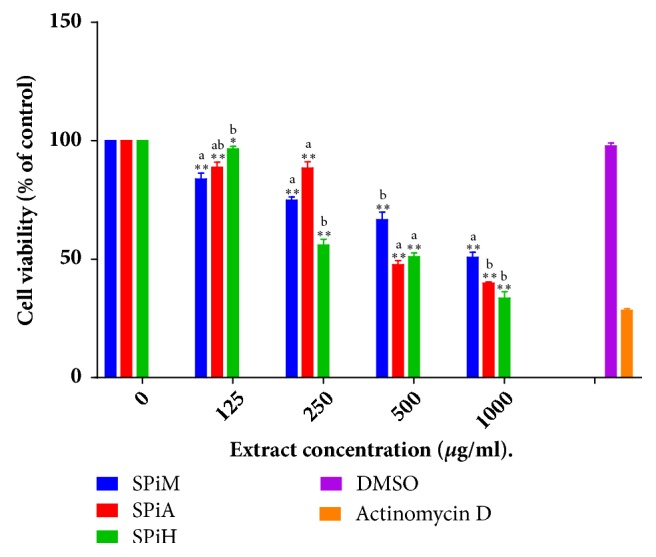
The effects of different extract concentrations of* S. pinnata *on the proliferation of murine myoblast cells (C2C12) as a percentage of the untreated control is represented as concentration versus cell viability. SPiM=*S. pinnata *methanol extract, SPiA=*S. pinnata *acetone extract, SPiH=*S. pinnata* hexane extract, and DMSO= Dimethylsulphoxide. The results were obtained from three independent experiments and expressed as means ± standard deviation. The statistical significance of the results was tested using One-Way Analysis of Variance (ANOVA) employing the Dunnett Multiple Comparisons Test. The* p* value significance was represented by an asterisk (*∗*) for* p* <0,05 and two asterisks (*∗∗*) for* p*<0,01. Superscripts  ^a,  b^ represent significant differences among the extracts at given concentrations. Extracts with the same superscript were not significantly different from each other while those with different superscripts showed significance of* p* <0,05.

**Figure 6:**
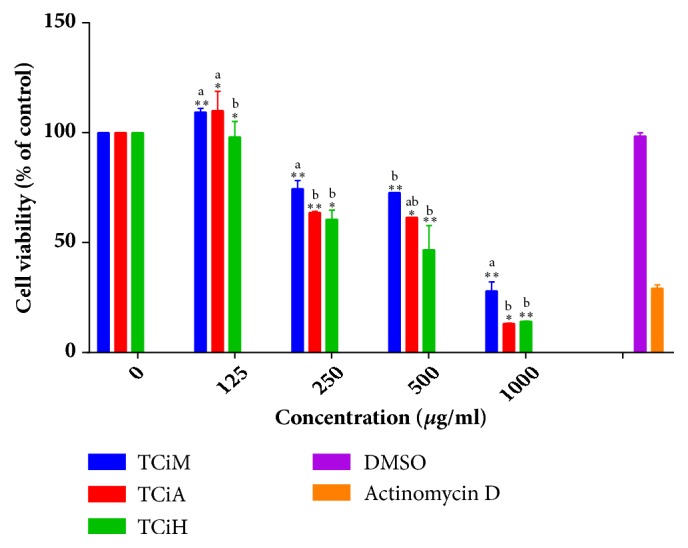
The effects of different extract concentrations of* T. ciliata *on the proliferation of murine myoblast cells (C2C12) as a percentage of the untreated control is represented as concentration versus cell viability. TCiM=*T. ciliata methanol* extract, TCiA=*T. ciliata* acetone extract, TCiH=*T. ciliata* hexane extract, and DMSO= Dimethylsulphoxide. The results were obtained from three independent experiments and expressed as means ± standard deviation. The statistical significance of the results was tested using One-Way Analysis of Variance (ANOVA) employing the Dunnett Multiple Comparisons Test. The* p* value significance was represented by an asterisk (*∗*) for* p*<0,05 and two asterisks (*∗∗*) for* p*<0,01. Superscripts  ^a,  b^ represent significant differences among the extracts at given concentrations. Extracts with the same superscript were not significantly different from each other while those with different superscripts showed significance of* p* <0,05.

The viability of C2C12 cell line was assessed at increasing concentrations of the different extracts using the MTT cell viability assay (Figures 5 and 6). The cell viability decreased as the concentration of the various extracts was increased. The different concentrations of* S. pinnata *extracts resulted in concentration dependant viability of cells after 24 hours of treatment. The highest concentrations of the methanol, acetone, and hexane extracts of* S. pinnata *resulted in 50.77%, 39.91%, and 33.52% cell viability, respectively, while the lowest concentrations of the same extracts resulted in 84.00%, 88.75%, and 96.49% cell viability (Figure 5).

The hexane extract of* T. ciliata *had the least CC_50_ value of 402.16 *μ*g/ml and was therefore concluded to be the most cytotoxic extract in this study, although the lowest concentration of the hexane extract was however noncytotoxic as it resulted in 98.07% cell viability (Figure 6).

### 3.7. Glucose Uptake Assay

**Figure 7:**
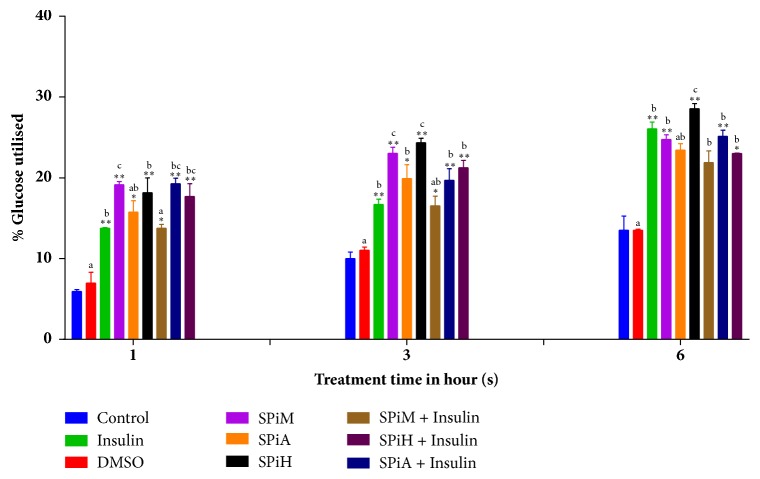
The effects of different extracts of* S. pinnata *at 100 *μ*g/ml in the presence or absence of insulin on the glucose uptake by murine myoblast cells (C2C12) at different time intervals. SPiM=*S. pinnata *methanol extract, SPiA=*S. pinnata *acetone extract, SPiH=*S. pinnata* hexane extract, and DMSO= Dimethylsulphoxide. The results were obtained from three independent experiments and expressed as means ± standard deviation. The statistical significance of the results was tested using One-Way Analysis of Variance (ANOVA) employing the Dunnett Multiple Comparisons Test between the control and the different treatments within the same time group. The* p* value significance was represented by an asterisk (*∗*) for* p*<0,05 and two asterisks (*∗∗*) for* p*<0,01.Superscripts  ^a,  b  and  c^ represent significant differences among the extracts at given time-points. Extracts with the same superscript were not significantly different while those with different superscripts showed significance of* p* <0,05.

**Figure 8:**
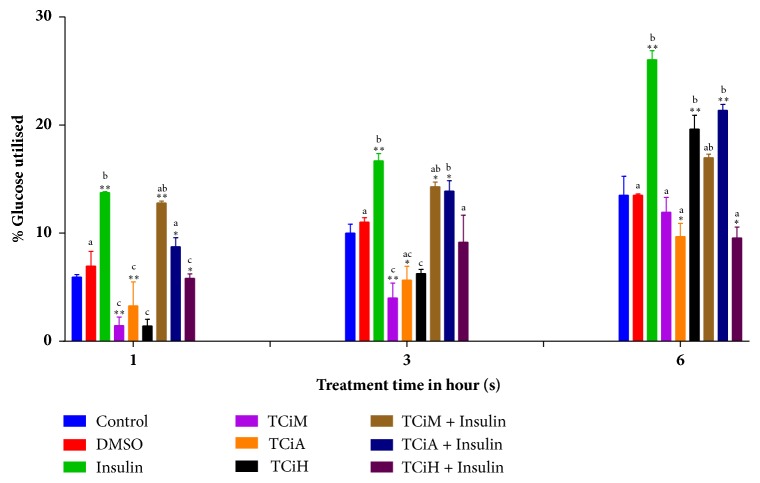
The effects of different extract of* T. ciliata *at 100 *μ*g/ml in the presence or absence of insulin on the glucose uptake by murine myoblast cells (C2C12) at different time intervals. TCiM=*T. ciliata *methanol extract, TCiA=*T. ciliata *acetone extract, TCiH=*T. ciliata *hexane extract, and DMSO= Dimethylsulphoxide. The results were obtained from three independent experiments and expressed as means ± standard deviation. The statistical significance of the results was tested using One-Way Analysis of Variance (ANOVA) employing the Dunnett Multiple Comparisons Test between the control and the different treatments within the same time group. The* p* value significance was represented by an asterisk (*∗*) for* p*<0,05 and two asterisks (*∗∗*) for* p*<0,01. Superscripts  ^a,  b  and  c^ represent significant differences among the extracts. Extracts with the same superscript were not significantly different while those with different superscripts showed significance of* p* <0,05.

Glucose utilisation by the differentiated C2C12 cells exposed to the different extracts was quantified by the glucose uptake assay (Figures 7 and 8). The percentage glucose utilised was calculated with respect to the untreated control at 1, 3, and 6 hours. The percentage glucose utilisation was observed to be lowest after 1 hour of incubation and highest after 6-hour incubation period for both plants.

All the extracts of* S. pinnata,* whether or not in combination with insulin, resulted in significant glucose utilisation at all treatment intervals, except for the acetone and methanol extract in combination with insulin at 6 hours (Figure 7). Individual treatments resulted in slightly high glucose utilisation above that of insulin at all treatment intervals, with the hexane extract being the most consistent, and it had the overall highest glucose uptake at 6 hours. Compared to insulin, a combination of insulin and the methanol extract had an overall poor glucose utilisation.

On the other hand, none of the treatments with the different extract of* T. ciliata *was able to increase glucose utilisation beyond that of insulin for all the time points (Figure 8). The highest glucose utilisation was shown for the acetone extract in combination with insulin which resulted in 21.37% glucose utilisation after 6 hours (Figure 8). This was closely followed by treatment with the hexane extract which had a glucose utilisation of 19.63%, followed by that of the methanol extract with 16.98% (Figure 8).

## 4. Discussion

Traditional medicinal plants exert various therapeutic effects against numerous ailments including diabetes mellitus through different mechanisms [26, 27]. With respect to diabetes, such mechanisms include, but are not limited to, insulin mimicking, insulin sensitising, alpha and beta glucosidase enzyme inhibition, reduction of glucose uptake by small intestine, and increasing glucose intake by peripheral cells [28–30]. The eventual goal of all these medications is to control the blood glucose level so that it remains within a nonpathological range. In this study, the ability of the different plant extracts to increase the uptake of glucose in C2C12 murine muscle cells as the peripheral cell model was examined. In addition, the antiglycation, antioxidant, and possible cytotoxic effects of the extracts were also independently assessed.

*Toona ciliata* and* S. pinnata* leaves were exhaustively extracted using methanol, acetone, and hexane. Plant sample preparation is a routine but critical first step in any medicinal plant study as it ensures the success of subsequent experiments [31]. It was observed that, in both plants, methanol which was the most polar solvent had the highest percentage extract yield and hexane the less polar solvent had the least.

Similar trends in which extraction profiles are polarity dependent have been observed in previous studies [31–33]. This trend may generally be attributed to polar constituencies being more abundant than nonpolar constituents in most plant leaf material [34]. It can on the other hand also be attributed to the small molecular size of methanol which may enable it to better penetrate the plant material resulting in higher extraction proficiency. On an overall basis, extracts of* T. ciliata* resulted in higher percentage yields compared to* S. pinnata,* most probably due to the highly fibrous nature of* S. pinnata* leaves as opposed to that of* T. ciliata*. Highly fibrous plant material contains more nonsoluble constituencies such as cellulose and pectin which in any case exhibit minimal therapeutic functions [35]. Further qualitative experiments were then carried out to determine the type of secondary metabolites that are present in the plant extracts.

The presence or absence of different phytocompounds that are mostly used as templates in different pharmacological therapeutic agents was determined. This is very important as to give an insight into the nature of the compounds that maybe responsible for the observed bioactivities [36, 37]. It should however be noted that compounds contained in plant extracts may work synergistically or even antagonistically [38]. Promising groups of phytocompounds that were detected in the selected plants with beneficial bioactivities observed in this study include phenols, flavonoids, and tannins [39].

Following the quantification of both phenols and flavonoids, both plants contained relatively higher amounts of phenols as compared to flavonoids in all extracts. Furthermore, all the* T. ciliata *extracts possessed more total phenols and flavonoids compared to* S. pinnata *extracts. These compounds have been shown to exert numerous therapeutic effects against inflammation, cancer, and diabetes and have been confirmed by several reports to be natural harbours of antioxidants [26, 39–41].

Compounds with antioxidant activity are valuable as they help reduce cellular damage that may result from oxidative stress within the body which formulates the initial pathogenic stage of many diseases [42–45]. Oxidative stress is an imbalance between the production of reactive species (free radicals) and antioxidant defences [46, 47]. Antioxidant compounds exert their therapeutic function by reacting with these reactive species (via electron or proton donation) or chelating them, thereby preventing them from reacting with and damaging functional biomolecules [48].* Toona ciliata* acetone extract exhibited the most potent DPPH radical scavenging activity with the lowest EC_50_ value of 1.90 mg/ml. The same extract equally exhibited similar potential (EC_50_ =5.26 mg/ml) in its ability to reduce ferric ion to its ferrous state, which further buttresses its antioxidant capability. The obtained EC_50_ values were however not lower than those for ascorbic acid which were 1.62 mg/ml and 3.10 mg/ml for the DPPH scavenging activity and the ferric reducing power assay, respectively. This may be due to the fact that the plant extract may contain inherent compounds that are emasculating the activity of the functional antioxidants [49, 50]. The observed antioxidant activities exhibited by the extracts of* T. ciliata* and* S. pinnata* can be related to the presence of phenols, flavonoids, terpenoids, and tannins [14, 51, 52]. The potency of this extract as an antioxidant agent may therefore be enhanced by further isolation and purification of the actual active compound [49, 50, 53].

Glycation, a spontaneous reaction between sugars and proteins, has also been implicated as a source of oxidative stress within the body which is the primary route for the formation of advanced glycation end-products (AGEs) [54]. Advanced glycation end-products have been implicated in the pathogenesis of diabetic complications. These products are eventually disposed from circulation through the kidney [5]. Consequently, AGEs put an extra strain on kidneys of diabetic patient's since they have accelerated rates of formation of AGEs due to perpetual hyperglycaemia. This may therefore culminate in nephropathy that is quite common among diabetic patients [55]. Hence pharmacological agents that reduce the glycation process are of importance in the management of diabetes and its complications including diabetic nephropathy. In this study it was observed that* T. ciliata* extracts resulted in the reduction of the glycation of BSA. Treatments with the methanol, acetone, and hexane extract of this plant resulted in 2,49%, 2,79%, and 2,56% glycation, respectively, which were more potent than the positive control, Arbutin, a known antiglycation agent. On the other hand, only the methanol extract of* S. pinnata* showed promise as an antiglycation agent. Antiglycation agents can help in maintaining the proper function and structure of molecules that would have otherwise been rendered nonfunctional through the spontaneous Millard reaction [56]. To the best of our knowledge, this study reports the antiglycation potential of both* S. pinnata* and* T. ciliata* for the first time.

While medicinal plants are recognised as a cheap form of therapeutic source with relatively good efficacy, their safety remains a worrisome cause for concern [57, 58]. This study thus evaluated the cytotoxic effect of extracts of* T. ciliata *and* S. pinnata* on C2C12 murine fibroblast cells using the MTT cell viability assay. The hexane extract of* T. ciliata *resulted in the lowest CC_50_ value of 402,16 *μ*g/ml. The lowest concentration of 125 *μ*g/ml of the hexane extract tested in the cytotoxic assay resulted in 98,07% cell viability and thus concentration below this cut-off was therefore employed in subsequent experiments together with the methanol and acetone extracts.

Glucose uptake from the bloodstream by peripheral cells helps reduce postprandial blood glucose levels, which in turn maintains normal glycaemic levels [59, 60]. In this study, C2C12 murine fibroblast cells were used as an* in vitro* model for muscle cells. The amount of glucose utilised by the differentiated C2C12 cells exposed to different treatment conditions was observed to increase over time indicating a cumulative glucose uptake activity. This was also evident for* T. ciliata* in a previous study [17], where the plasma glucose level in Streptozotocin (STZ) induced diabetic rats was significantly reduced when treated with a hydroalcoholic extract for two weeks. Despite* T. ciliata* showing increasing glucose utilisation overtime, its overall glucose utilisation potential was very poor when compared to that of insulin at all-time intervals. In this study, the hexane extract of* S*.* pinnata* resulted in enhanced glucose uptake which supersedes that of insulin at 6 hours. These results are similar to our previous study that indicated the n-hexane extract of* S. plumosum *exhibited better glucose utilisation compared to insulin [61]. This might suggest that nonpolar compounds may be more capable of modulating glucose uptake in C2C12 cells compared to polar compounds. Furthermore, the high glucose uptake ability of the hexane extract of* S. pinnata* by the C2C12 muscle cells above that of insulin observed in this study is contrary to the findings of Deutschländer [62]. In that study glucose utilisation by C2C12 muscle cells was reported to be poor as opposed to that of 3T3-L1 adipocytes and Chang liver cells treated with ethanol extract of* S. pinnata* in comparison to insulin. The reason for the different observations between these studies may be the solvent types used for extraction in the studies and possible variations in the amounts of biologically active phytotherapeutic agents in the extracts. Insulin is known to elicit the highest glucose uptake in peripheral cells and as a result facilitates the maintenance of euglycemic levels. Therefore, any extract that elicits a similar or more pronounced response should be considered as a potential source of agents with immense hypoglycaemic therapeutic potential for further isolation and characterisation of such agents.

## 5. Conclusion

The study highlights the potential of the different plant extracts with respect to the various parameters evaluated. The different bioactivities observed may be mainly attributed to different phytoconstituencies within the different extracts. Overall,* T. ciliata* extracts showed the highest antiglycation potential, with the acetone extract having potent antioxidant constituents. The hexane extract of* S. pinnata* that had the least extraction yield and total phenolic and flavonoid content showed the most potent glucose uptake ability in this study. Work is in process to isolate and identify these active compound(s) within the extracts that exhibited potent antioxidant, antiglycation, and hypoglycaemic effects.

## Figures and Tables

**Table 1:** The presence/absence of various secondary metabolites in the different crude plant extracts of the different solvents.

Phytochemicals	Extracts					
	SPiM	SPiA	SPiH	TCiM	TCiA	TCiH
**Tannins**	+	+	+	+	+	+
**Flavonoids**	+	+	-	+	+	+
**Phenols**	+	+	+	+	+	+
**Saponins**	-	-	-	-	-	-
**Steroids**	+	+	-	+	+	-
**Phlobatannins**	-	-	-	-	-	-
**Glycosides**	+	+	-	-	-	-
**Coumarins**	+	+	-	+	+	+
**Proteins**	+	+	-	-	-	-
**Anthraquinones**	-	-	-	-	-	-
**Anthocyanins**	-	-	-	-	-	-
**Leucoanthocyanins turns**	-	-	-	-	-	-
**Carbohydrates**	-	-	-	-	-	-

(-) = constituent absent; (+) = constituent present. SPiM=*S. pinnata *methanol extract, SPiA=*S. pinnata *acetone extract, SPiH=*S. pinnata* hexane extract, TCiM=*T. ciliata *methanol extract, TCiA=*T. ciliata* acetone extract, and TCiH=*T. ciliata* hexane extract

**Table 2:** The EC_50_ values for the DPPH scavenging assay and the ferric reducing power of the *T. ciliata *and *S. pinnata* extract.

Extract	DPPH scavenging assay EC_50_ (mg/ml)	Ferric reducing power assay EC_50_ (mg/ml)
SPiM	10.32±0.005*∗*^a^	11.82±0.054^a^
SPiA	11.30±0.011*∗∗*^a^	12.68±0.074*∗*^a^
SPiH	9.52±0.041^a^	18.74±0.004*∗∗*^a^
TCiM	2.36±0.002*∗∗*^b^	6.27±0.047*∗*^b^
TCiA	1.90±0.016*∗∗∗*^b^	5.26±0.001*∗∗*^a^
TCiH	7.86±0.044^a^	10.21±0.047^a^
Ascorbic acid	1.62±0.007	3.10±0.083

SPiM=*S. pinnata *methanol extract, SPiA=*S. pinnata *acetone extract, SPiH=*S. pinnata* hexane extract, TCiM=*T. ciliata *methanol extract, TCiA=*T. ciliata *acetone extract, and TCiH=*T. ciliata* hexane extract. Superscripts  ^a,  b^ represent significant differences among the extracts. Extracts with the same superscript were not significantly different while those with different superscripts showed significance of *p* <0,05. The asterisk represents significant difference between the extracts and the positive control. The *p* value significance was represented by an asterisk (*∗*) for *p* <0,05, two asterisks (*∗∗*) for *p* <0,01, and three asterisks (*∗∗∗*) for *p* <0,001.

## Data Availability

The data used to support the findings of this study are available from the corresponding author upon request.
